# Bionomics of the malaria vector *Anopheles farauti *in Temotu Province, Solomon Islands: issues for malaria elimination

**DOI:** 10.1186/1475-2875-10-133

**Published:** 2011-05-18

**Authors:** Hugo Bugoro, Robert D Cooper, Charles Butafa, Charles Iro'ofa, Donna O Mackenzie, Cheng-Chen Chen, Tanya L Russell

**Affiliations:** 1 National Vector Borne Disease Control Programme, Ministry of Health, Honiara, Solomon Islands; 2 Institute of Tropical Medicine, National Yang-Ming University, No. 155, Sec.2, Li-Nong Street, Taipei 112, Taiwan; 3 Australian Army Malaria Institute, Gallipoli Barracks, Enoggera, 4052, Australia; 4 The University of Queensland, School of Population Health, Australian Centre for Tropical and International Health, Pacific Malaria Initiative Support Centre, Herston, 4006, Australia

## Abstract

**Background:**

In the Solomon Islands, the Malaria Eradication Programmes of the 1970s virtually eliminated the malaria vectors: *Anopheles punctulatus *and *Anopheles koliensis*, both late night biting, endophagic species. However, the vector, *Anopheles farauti*, changed its behaviour to bite early in the evening outdoors. Thus, *An. farauti *mosquitoes were able to avoid insecticide exposure and still maintain transmission. Thirty years on and the Solomon Islands are planning for intensified malaria control and localized elimination; but little is currently known about the behaviour of the vectors and how they will respond to intensified control.

**Methods:**

In the elimination area, Temotu Province, standard entomological collection methods were conducted in typical coastal villages to determine the vector, its ecology, biting density, behaviour, longevity, and vector efficacy. These vector surveys were conducted pre-intervention and post-intervention following indoor residual spraying and distribution of long-lasting insecticidal nets.

**Results:**

*Anopheles farauti *was the only anopheline in Temotu Province. In 2008 (pre-intervention), this species occurred in moderate to high densities (19.5-78.5 bites/person/night) and expressed a tendency to bite outdoors, early in the night (peak biting time 6-8 pm). Surveys post intervention showed that there was little, if any, reduction in biting densities and no reduction in the longevity of the vector population. After adjusting for human behaviour, indoor biting was reduced from 57% pre-intervention to 40% post-intervention.

**Conclusion:**

In an effort to learn from historical mistakes and develop successful elimination programmes, there is a need for implementing complimentary vector control tools that can target exophagic and early biting vectors. Intensified indoor residual spraying and long-lasting insecticide net use has further promoted the early, outdoor feeding behaviour of *An. farauti *in the Solomon Islands. Consequently, the effectiveness of IRS and the personal protection provided by bed nets is compromised. To achieve elimination, any residual transmission should be targeted using integrated vector control incorporating complementary tools such as larviciding and/or zooprophylaxis.

## Background

The leading vector control tools, long-lasting insecticidal nets (LLINs) and indoor residual spraying (IRS) can be very effective at reducing malaria transmission [1-4]. The efficacies of these vector control tools depends heavily of the behaviour of the malaria vectors, and are more effective against vectors which bite indoors (endophagic), late in the night (nocturnal) and which rest indoors after feeding (endophilic). These characteristics are classically observed for the primary malaria vectors in sub-Saharan Africa and are contributing to the success of the malaria programmes in this region [1,2,5,6]. However in the southwest Pacific, the feeding and resting behaviour of vectors are much more variable, with some species expressing a tendency to bite early in the night, outdoors (exophagic), on animals (zoophagic) and to rest outdoor (exophilic) [7-9]. Such behaviours could reduce the efficacy of vector control tools which rely on vectors feeding indoors.

The malaria eradication programmes of the 1960-1970s were not successful due to a number of factors including logistical and financial limitations as well as technical and operational issues [10]. With regards to the vector, insecticide resistance was a major world-wide problem [11,12]. However, in the southwest Pacific, there has been no evidence of physiological insecticide resistance despite decades of DDT-IRS followed by pyrethroid-IRS and treated nets. What has been observed, particularly in one of the major vectors, *Anopheles farauti*, is a change of biting behaviour. In Papua New Guinea, the Solomon Islands and Vanuatu it was found that there was a shift in the behaviour of this vector from feeding indoors late in the night to outdoors early in the night [8,13-16]. This change in behaviour was most likely due to vector irritability caused by the DDT leading to the vector avoiding insecticide exposure [5,14]. In the Solomon Islands, it was found that the proportion of *An. farauti *biting from 6-8 pm increased from 30% before IRS to 66% after IRS; also the proportion biting indoors changed from 53% before IRS to 33% after IRS [8]. Another factor, relevant to the efficacy of IRS, was that the excito-repellent effect of the DDT reduced or precluded the time *An. farauti *spent indoors resting so that at least 45% of *An. farauti *exited houses without picking up a lethal dose of insecticide [16]. This change in resting and biting behaviour reduced the level of personal and communal protection afforded by IRS and ultimately led to the failure of the eradication programme despite the addition of mass drug administration to the programme [17,18].

The international community recently prioritized national and regional elimination with a long term goal of malaria eradication, based on a key strategy of shrinking the malaria map from the margins inwards [19]. Worldwide, a number of geographically defined regions have adopted the goal of regional malaria elimination, including Temotu Province in the Solomon Islands. Temotu Province was selected for elimination because, in addition to its geographic isolation, the incidence of malaria has remained low since the initial eradication efforts in the 1970s. When the current elimination program commenced in 2008, the overall parasite rate determined by mass blood survey was estimated to be 3.83% [20]. The only anopheline found in the Province has been *An. farauti *s.l. [21]; however more recent molecular-based studies have identified three species of the Farauti Complex in the Solomon Islands [22,23]. Allozyme electrophoresis was used to identify *An. farauti *from Ndendo [22] and while it is most likely that this is the only species on the other islands this is yet to be confirmed.

Given the history of *An. farauti*, its reaction to IRS, and that the primary intervention measures intended for the elimination of malaria in Temotu Province will target the vector by IRS or LLINs, it is essential to understand the entomological consequences of wide-spread and long-term insecticide pressure. Therefore, the vector population was studied to determine its pre-intervention behaviour and up to 12 months following the implementation of intervention measures. It was hypothesized that further wide-scale use of insecticides through LLIN and IRS will reinforce the early outdoor feeding behaviour of these vectors.

## Methods

### Study site

**Figure 1:**
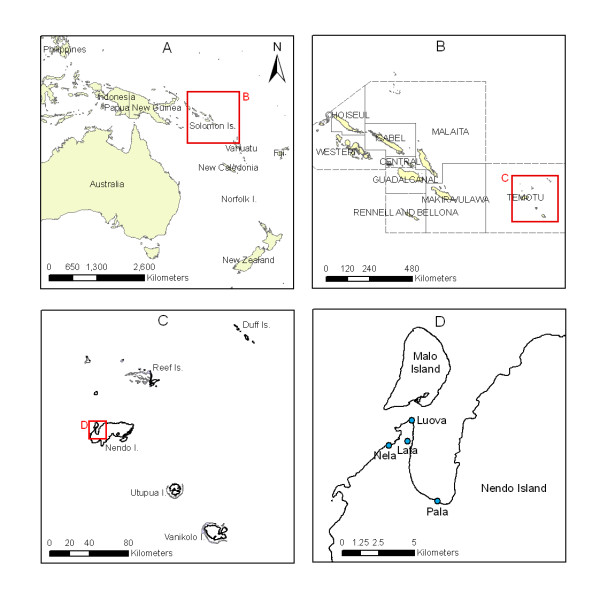
**Map of the southwest Pacific Region (A) showing the Solomon Islands (B), Temotu Province (C) and Nendo Island (D; 10°42'S, 165°48'S)**.

The study was conducted in Temotu Province, the eastern most province of the Solomon Islands (10°42'S, 165°48'S; Figure 1). The province is made up of five main islands: Ndendo, Reef, Duff, Utupua, and Vanikoro. The Reef Islands are low lying coral formations while all the other islands are volcanic in origin and consist of a narrow coastal shelf rising steeply to a rugged mountainous interior. Only on the eastern end of Ndendo Is., is there a level inland on the plateau region about 150 m above sea level. Ndendo is the largest island, approximately 675 km^2^, it is the most populated and contains the provincial capital, Lata. The community villages are located almost exclusively along the coast with only a few villages being located inland the plateau on Ndendo Island.

**Figure 2:**
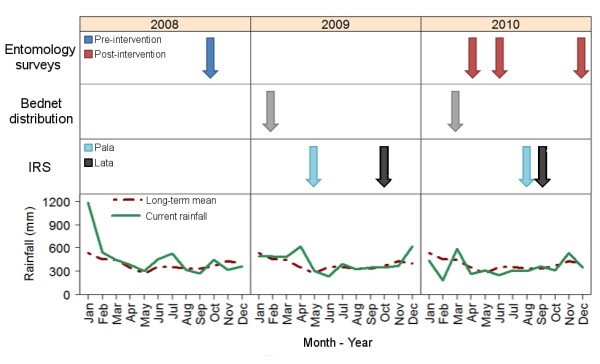
**The timeline of entomological surveys and vector control activities as implemented during this study as well as contextual rainfall**.

The climate is continually hot and wet with a median annual rainfall of 4,297 mm (Figure 2). There is no clear dry season, although during April to September the rainfall is slightly lower (range: 290 mm - 336 mm) than the average monthly rainfall (345 mm). During the wetter months, periods of more than four days without rain are rare. The mean annual maximum and minimum temperatures are 30.15°C and 24.14°C respectively.

### Experimental design

Implementation of the current elimination programme began in Temotu Province in late 2008. Free LLIN (active ingredient: deltamethrin; NetProtect^®^) were provided to all householders with distribution commencing in February-March 2009 and in March 2010 (Figure 2). IRS (active ingredient: lambda-cyhalothrin; formulation: ICON^® ^10CS) was conducted in all houses within 2 km of the coastline. The survey village, Pala, was sprayed in May 2009 and August 2010 while Lata was sprayed in October 2009 and September 2010. Prior to the commencement of the elimination programme, pre-intervention entomological surveys were conducted in October 2008. Post-intervention surveys were conducted in April, June, and December 2010 (Figure 2). The aim was to compare changes in baseline entomological parameters after intensive vector control operations.

### Larval surveys

To provide baseline information about the distribution of mosquitoes within the Province, larval surveys using standard 250 ml dippers were conducted on Ndendo, Reef, Duff, Vanikoro and Utupua Islands (Figure 1C). The surveys were primarily along the coast, though on eastern Ndendo they extended inland onto the plateau region. Larval habitats were described and their location recorded by GPS and marked on 1:50,000 scale topographic maps.

### Human landing catches

During October 2008, at the start of the elimination drive, human landing catches (HLC) were conducted from 6 pm - 6 am in four villages on Ndendo Island: Pala (5 nights), Lata (10 nights), Nelo (1 night) and Luava (1 night) (Figure 1D) as well as Ghauta village on the Duff Islands. Following the implementation of the elimination measures further HLC were made in Lata during April 2010 (1 night) and in Pala during April (5 nights), June (4 nights) and December (4 nights) 2010. During the April and December surveys mosquitoes were collected from 6 pm - 6 am, during the June survey mosquitoes were collected over the peak biting period 6 pm - 10 pm.

All human landing catches (HLC) were conducted by six collectors, three indoors and three outdoors, all mosquitoes coming to bite their exposed feet and legs were caught using an aspirator for 40 min each hour. The first team of six collectors worked from 6 pm to midnight and the second team of six collectors worked from midnight to 6 am. Mosquitoes were held in individual waxed paper cups for each hour, location -(indoor or outdoor) and household. The following morning, mosquitoes were killed, identified by morphology [24], counted, and dissected to determine parity [25].

### Longevity of the vector populations

The ovaries of mosquitoes caught in the night landing catches were dissected in physiological saline transferred to water, allowed to dry and examined under 100-200X for the presence or absence of skeins at the end of the trachea [25]. From this the proportion parous (P) was used to determined the survival through one day (*p*) as *p *= ^*x*^√P; where *x *is the length of the gonotrophic cycle. The proportion of the vector population living long enough to transmit malaria was determined by *p*^*n*^; where *p *is the survival through one day (from above) and *n *is the extrinsic incubation period. The extrinsic incubation period is 9 days for *Plasmodium vivax *and 12 days for *Plasmodium falciparum *[26].

### Human host movement

During the 2008 survey in both Lata and Pala, the number of people in the village that were outdoors was counted each hour from 6 pm to 6 am, this was done on each night of the HLC. A census was conducted of each village to determine the entire population and thus the number of people indoor and outdoor each hour was determined.

### Estimating human contact with mosquito bites occurring indoors

The biting behaviour of *An. farauti *in Pala was compared using: 1) propensity to bite indoors (endophagy); 2) propensity to bite during sleeping hours (nocturnal activity); and 3) the proportion of human contact with mosquito bites occurring outdoors (π_i_). The degree of endophagy was calculated as the proportion of mosquitoes biting indoors as follows: *I*_6 pm_→_5 am_/(*I*_6 pm_→_5 am _+ *O*_6 pm_→_5 am_); where *I *= the total number of mosquitoes caught indoors, *O *= the total number of mosquitoes caught outdoors and the subscripts represent the start time for each hour [27]. Nocturnal activity was calculated as the proportion of mosquitoes biting either indoors or outdoors during peak sleeping hours (hours starting 9 pm to 4 am; see Results Table 1) as follows: (*I*_9 pm_→_4 am _+ *O*_9 pm_→_4 am_)/(*I*_6 pm_→_5 am _+ *O*_6 pm_→_5 am_) [27]. The proportion of human contact with mosquito bites that occurs indoors, provides an indication of the efficacy of the current vector control tools which targeting these indoor biting mosquitoes which has been adjusted for the bias created by the location of the collector and the movement of village people in doors during the night. The proportion of indoor human contact with mosquito bites (π_i_) was calculated by multiplying the number of *An. farauti *biting indoors with the number of people indoors for each hour of the night and similarly the outdoor biting component (π_o_) was multiplied by the number of people outdoors for each hour of the night: *π*_*i *_= ∑[*I*_*t*_*S*_*t*_]/∑[*O*_*t*_(1-*S*_*t*_)] + *I*_*t*_*S*_*t*_; where *S *= the proportion of humans indoors [see [28] for more detail]. Statistical changes in endophagy, nocturnal activity and the estimate of human contact were compared over time using generalized linear mixed models (GLMM) with a binomial distribution, a categorical explanatory variable for study period and random factors for date and household. All analyses were conducted using the *R *package V.2.9.1.

**Table 1:** The percentage of the human population indoors throughout the night in Lata and Pala villages, Ndendo Island, Temotu Province, Solomon Islands.

Hour	Lata	Pala
6-7 pm	7.9	14.6
7-8 pm	39.8	28.3
8-9 pm	72.2	70.2
9-10 pm	88.3	80.0
10-11 pm	90.1	83.4
11-12 pm	93.6	91.2
12-1 am	91.5	96.6
1-2 am	100	93.6
2-3 am	99.7	100
3-4 am	100	100
4-5 am	99.7	100
5-6 am	87.8	55.1

**Total population**	**38**	**41**

### Length of the gonotrophic cycle

The length of the gonotrophic cycle was measured directly from mosquitoes, which were blood-fed immediately after capture. A total of 40 mosquitoes, caught by HLC during 6-7 pm, were bloodfed and placed individually into 70 ml specimen jars. The bottom of each jar contained a wad of damp cotton-wool covered with filter paper; the top of each jar was covered with netting overlayed with damp cotton-wool. After 48 hours each container was examined hourly and the time and number of eggs laid was recorded.

### Mosquito identification and sporozoite detection

All specimens from the larval collections were preserved in 70% ethanol, while all adults collected were desiccated and preserved on silica gel. Sporozoite infection of the head and prothorax of adult mosquitoes was determined using ELISA [29,30]. Specimens were considered positive if the optical density was twice that of the negative control average (n = 5), all positives were rerun for confirmation. Identification of members of the *An. farauti *complex in Temotu was determined using polymerase chain reaction restriction fragment length polymorphism (PCR-RFLP). Up to 5 individual larvae from each larval collection site and adults from the HLC, Exit Window Traps (EWT) and indoor resting searches from the 2008 surveys were processed. DNA extraction, amplification, restriction digest, fragment separation, and visualisation are as previously described [31]. All molecular assays were performed at the Australian Army Malaria Institute.

### House resting and exiting behaviour

Six EWT were positioned in houses in Lata for five nights and then in Pala for 15 nights. The traps were emptied each morning between 6-7 am. The abdomens of collected mosquitoes were visually classified as unfed, bloodfed, half gravid or gravid. During April 2010, indoor resting mosquitoes were collected from three houses in Pala village on four occasions. The collections commenced at 6 am and using an aspirator and torch the vertical surfaces within each house were searched for 35 min for any resting mosquitoes. All mosquitoes collected were examined to determine whether they were unfed, bloodfed, half gravid or gravid.

### Alternative host availability

*Anopheles farauti *is an indiscriminate feeder that can be readily diverted to other hosts [15,32]. To understand the potential of this occurring, a census of the availability of alternative hosts--dogs and pigs--was carried out in Pala and Lata, during April 2010. Two pig pens in Pala village were also searched for resting mosquitoes on each of four mornings commencing at 6 am, searches were made for 35 minutes. For any mosquitoes collected the stomach and ovary condition was recorded.

### Ethics

Ethical approval for the study was obtained from the University of Queensland Medical Research Ethics Committee (2010000412).

## Results

### Anopheles species composition and distribution

On morphological examination, all adult anophelines were *An. farauti *s.l.. Molecular identification (n = 280 PCR amplifications) of specimens from larval collections made throughout the Province and adult collections from Ndendo revealed that the only anopheline in the Province was *An. farauti s.s*. On Ndendo Island, which is the only island with a sizable inland area, *An. farauti *was found in larval habitats and biting humans up to 5 km inland at altitudes of >160 m.

### Larval ecology

*Anopheles farauti *larvae were collected from 27 larval sites throughout the Province. Sites were either human made or naturally occurring. The range of smaller sites included temporary ground pools, such as wheel ruts, pools in earthen drains and borrow-pits associated with road works, natural depressions and spring wells, the larger sites included coastal lagoons and swamps of over a hectare. The smaller sites were generally maintained by regular rainfall. Both brackish and fresh water sites were used. Most sites were well established with aquatic vegetation, emergent grasses and algae. Around the indicator villages of Pala and Lata, the temporary ground pools were too small and not numerous enough to account for the high adult densities recorded in these villages. However, both Pala and Lata are located within 1 km of large coastal swamps and it appears that these sites are responsible for maintaining the high biting densities. Nela village was also co-located with a swamp but this was dry at the time of these surveys. On the Duff Islands, Ghauta village was located close to a swamp.

### Biting density

The biting density varied considerably between the different villages surveyed (Table 2). On Ndendo Is. the villages Pala, Lata, Nela and Luava were all within 5 km of each other, yet the landing densities during October 2008 ranged from high at Pala (78.5 bites/person/night [b/p/n]), moderate at Lata (19.5 b/p/n) to low at Nelo (2.42 b/p/n) and Luava (0.5 b/p/n). On the Duff Is., at Ghauta village, the landing rate was 37 b/p/n. This heterogeneity in biting densities was associated with the large, productive breeding sites (coastal swamps) co-located with Pala, Lata and Ghauta villages. At Pala, the five nights of HLC were made over 25 days and at Lata, the 10 nights of HLC were made over 20 days. Over this period the biting numbers were fairly stable with no rapid rises and crashes in population densities. Post-intervention (April 2010), no *An. farauti *were collected in Lata; however this was most likely due to the natural drying of the large swamp which was the main productive larval site. At Pala the nearby swamp remained productive and the overall post-intervention biting rate was 22.6 b/p/n in April and 62.29 b/p/n in December 2010 (Table 2). Additional collections made in late June 2010 in Pala from 6 pm - 10 pm and over four nights 269 *An. farauti *were collected with a reasonably high overall hourly biting rate of 11.19 b/p/h for this four-hour period (Table 3).

**Table 2:** The entomological estimation of malaria transmission intensity attributable to *Anopheles farauti *on Ndendo Island, Temotu Province, Solomon Islands during 2008 and 2010.

Entomological parameters	Pre-intervention		Post-intervention		
	
	Lata Nov 2008	Pala Nov 2008	Pala Apr 2010	Pala June 2010	Pala Dec 2010
Sporozoite rate (S) (*n *= number assayed)	0.0026 (*n *= 1,150)	0.0000 (*n = *2,561)	0.0013 (*n = *749)	0.0015 (*n *= 1,308)	0.0008 (*n *= 1,292)

Biting rate (B; b/p/n)				NI	
Indoor	8.56	68.54	10.13		44.50
Outdoor	30.42	88.47	35.13		80.08
Overall	19.5	78.5	22.63		62.29

Entomological inoculation rate (EIR; ib/p/y)	18.25	<0.001	10.74	NI	17.59

Endophagy (Proportion indoors ± se)	0.221 ± 0.012 (*n *= 1,172)	0.432 ± 0.010 (*n *= 2,355)	0.223 ± 0.016 (*n *= 679)	NI	0.357 ± 0.012 (*n *= 1,495)

Nocturnal biting (Proportion 9 pm-4 am ± se)	0.503 ± 0.014 (*n *= 1,172)	0.523 ± 0.014 (*n *= 2,355)	0.546 ± 0.019 (*n *= 679)	NI	0.378 ± 0.012 (*n *= 1,495)

Proportion of indoor contact (π_i_)	0.368 ± 0.120	0.570 ± 0.059	0.367 ± 0.108	NI	0.404 ± 0.063

Where, S = no. of sporozoite positive mosquitoes/no. of mosquitoes tested; B = no. of mosquitoes collected/no. of nights/no. of collectors; and EIR = S × B_overall _× 365.

Endophagy is the proportion of mosquitoes caught indoors; nocturnal biting is the proportion of mosquitoes caught during hours when most people are asleep; and the proportion of indoor contact is the indoor and outdoor biting rates weighted throughout the night by the proportion of humans that are typically indoors or outdoors at each time period - see Methods for details. NI = data not included as HLC made from 6-10 pm.

**Table 3:** The mean hourly biting rate (± se) of *Anopheles farauti *on Ndendo Island, Temotu Province, Solomon Islands during 2008 and 2010 during 6 pm - 10 pm.

Biting rate (B; b/p/h)	Pre-intervention		Post-intervention		
	
	Lata Oct 2008	Pala Oct 2008	Pala Apr 2010	Pala Jun 2010	Pala Dec 2010
Indoor	1.27 ± 0.31	14.10 ± 4.69	1.06 ± 0.21	5.31 ± 1.31	7.04 ± 1.37
Outdoor	4.97 ± 0.85	11.50 ± 2.09	4.68 ± 0.59	17.06 ± 2.55	15.60 ± 3.15
Overall	3.12 ± 0.49	12.80 ± 2.54	2.87 ± 0.35	11.18 ± 1.56	11.32 ± 1.76

### Interaction between human hosts and vectors

**Figure 3:**
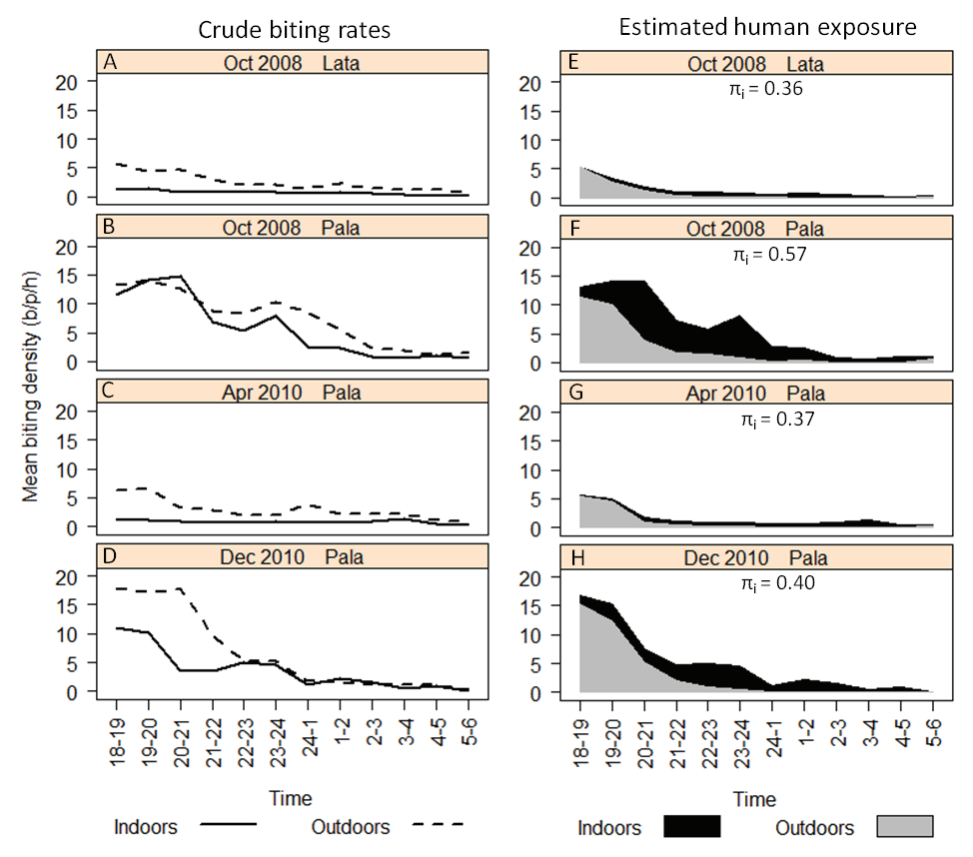
**The hourly indoor and outdoor profile of *Anopheles farauti *biting (A-D) and estimated human contact to these mosquito bites (E-H) in Lata and Pala villages, Ndendo Island, Temotu Province, Solomon Islands during 2008 and 2010**. Note: For E-H, the stacked line graph represents estimates of indoor contact take into consideration the movement pattern of people by weighting the indoor and outdoor biting rates throughout the night by the proportion of humans that are typically indoors or outdoors at each time period [28]; b/p/h = bites/person/hour.

At the commencement of the elimination campaign (2008) in Pala the *An. farauti *population was primarily exophagic with only 43.2% of biting occurring indoors. The peak biting time was 7-8 pm though half (52.3%) of biting occurring during sleeping hours (Figure 3). After the implementation of IRS and LLINs in Pala, there was evidence that the indoor biting proportion of the population had been reduced (*p *= 0.0008; Table 4). In the Dec 2010 survey, endophagy had slightly, but not significantly, been reduced to 35.7% (*p *= 0.225). Regarding nocturnal activity, the intervention measures appeared to enforce the early night biting activity of the vector (*p *= 0.0027) with the Dec 2010 survey indicating that the proportion biting during sleeping hours had been reduced to 37.8% (*p *= 0.0028; Table 4).

**Table 4:** The proportion of host-seeking *Anopheles farauti *caught indoors and during sleeping hours as well as the proportion of contact occurring indoors (π_i_) during 2008 and 2010 in Pala Village.

Month-Year	**Proportion ± s.e**.	n/N	Odds ratio [95% CI]	*p *value
***Endophagy***				
Oct 2008	0.432 ± 0.010	1,017/2,355	1.00	NA
Apr 2010	0.223 ± 0.016	152/679	0.551 [0.426 - 0.712]	<0.0001
Dec 2010	0.357 ± 0.012	534/1,495	0.873 [0.700 - 1.087]	0.362
Overall influence of time		1,703/4,529	NA	0.0008
***Nocturnality***				
Oct 2008	0.523 ± 0.014	1,103/2,355	1.00	NA
Apr 2010	0.546 ± 0.019	371/679	1.156 [0.983 - 1.360]	0.080
Dec 2010	0.378 ± 0.012	565/1,495	0.804 [0.697 - 0.928]	0.0028
Overall influence of time		2,039/4,529	NA	0.0027
***Proportion of indoor contact (π***_***i***_***)***				
Oct 2008	0.570 ± 0.059	602/1,070	1.00	NA
Apr 2010	0.367 ± 0.108	109/297	0.664 [0.509 - 0.865]	0.0024
Dec 2010	0.404 ± 0.063	295/731	0.727 [0.593 - 0.892]	0.0022
Overall influence of time		1,006/2,099	NA	0.0087

The proportions for each survey period are compared using generalized linear mixed models (GLMMs) with a binomial distribution, a categorical explanatory variable for study period and random factors for date and household; October 2008 formed the reference category for the GLMM.

Endophagy is the proportion of mosquitoes caught indoors; nocturnality is the proportion of mosquitoes caught during hours when most people are asleep; and indoor contact is the proportion of indoor and outdoor biting rates weighted throughout the night by the proportion of humans that are typically indoors or outdoors at each time period - see Methods for details.

The changes in entomological behaviour are epidemiologically relevant because the proportion of human contact occurring indoors (π_i_) was significantly reduced (*p *= 0.0087) following the intervention measures. Considering that the majority of the human population (71.7%; Table 1) were outdoors during the hours of peak biting activity, a high level of human contact with mosquitoes was facilitated outdoors. At the commencement of the campaign in 2008, 57.0% of all biting occurred indoors. However, by Apr 2010, π_i _had dropped to 36.7% (*p *= 0.0024) and by Dec 2010 was at 40.4% (*p *= 0.0022; Table 4). During the post-intervention surveys the vast bulk of contact with mosquito bites occurred outdoors, primarily before 9 pm (Figure 3).

### Duration of the gonotrophic cycle

Of the 40 *An. farauti *bloodfed and set up for oviposition, 34 survived with 28 (82.4%) laying on the second night (47-58 hours after blood feeding) and 6 (17.6%) laying on the third night (71 - 82 hours after blood feeding). The median time to oviposition was 59 hrs, thus the gonotrophic cycle was approximately 2.5 days. The mean number of eggs laid per female was 107 (range 17-277).

### Longevity of the *An. farauti *populations

Dissections to measure parity were made on 1,217 *An. farauti *collected at Pala and 842 *An. farauti *collected at Lata during October 2008. The proportion parous was similar for each hour at both Pala and Lata (Table 5). The only heterogeneity noted was for the 6-7 pm collection period where, for both Pala and Lata, the proportion parous was significantly lower than for the other hours of the night (χ^2 ^= 5.67, P < 0.05). The overall proportion parous was 0.416, for both Lata and Pala. The daily survival rate (*p*) of the *An. farauti *populations on Ndendo Island was calculated to be 0.704. As such, 1.5% of the population was living long enough to transmit *P. falciparum *and 2.99% of population was living long enough to transmit *P. vivax*. Post intervention surveys (April and December 2010) in Pala village, the proportion parous was 0.43 for both sample points (Table 5) which would indicate a daily survival rate of 0.713, similar to the 2008 estimates. There was no difference in the proportion parous at different hours of the night and for outdoor and indoor collections. Additional collections and dissection in June 2010 indicated a parous rate of 0.49 (n = 98).

**Table 5:** The proportion of parous mosquitoes within the *Anopheles farauti *population in Lata and Pala villages, Temotu Province, Solomon Islands, calculated from hourly mosquito collections during 2008 and 2010.

Hour	Pre-intervention		Post-intervention	
	
	Lata Oct 2008	Pala Oct 2008	Pala Apr 2010	Pala Dec 2010
6-7 pm	0.24	0.28	0.33	0.39
7-8 pm	0.39	0.40	0.36	0.45
8-9 pm	0.34	0.31	0.34	0.22
9-10 pm	0.36	0.48	0.40	0.46
10-11 pm	0.40	0.51	0.46	0.40
11-12 pm	0.52	0.48	0.56	0.50
12-1 am	0.39	0.43	0.50	0.50
1-2 am	0.66	0.45	0.53	0.50
2-3 am	0.47	0.47	0.52	0.44
3-4 am	0.60	0.26	0.49	0.20
4-5 am	0.45	0.51	0.58	0.44
5-6 am	0.62	0.48	0.64	NA

**Overall parity**	**0.41**	**0.42**	**0.43**	**0.43**

**Daily survival (*p*)**	**0.700**	**0.707**	**0.713**	**0.713**

**Total mosquitoes (*n*)**	**842**	**1,218**	**645**	**343**

Heterogeneity was noted in the proportion parous for *An. farauti *collected 6-7 pm as compared to the other hours of the night in Lata and Pala during Oct 2008 (χ^2 ^= 5.67, P > 0.05).

### House resting and exiting behaviour

The EWTs did not prove to be a highly effective tool for sampling mosquitoes, largely due to house construction. The houses were made from palm leaf or bamboo and contained numerous gaps in the walls and large unscreened windows that could be used as alternative exits. In Lata no *Anopheles *were collected in the EWTs while in Pala a total of 196 *An. farauti *were collected. The EWTs were more useful at Pala due to the prevailing high vector densities. Of those mosquitoes exiting the house 76.5% failed to obtain a blood meal either due to bed net use by the occupants or due to the irritability effect of the insecticide. Only unfed and blood fed mosquitoes were collected in the EWT, no half-gravid or gravid mosquitoes were collected. During April 2010, a total of 84 *An. farauti *were collected resting inside of houses, a mean of 7.00 (se ± 2.61) per house/night. The proportion of unfed mosquitoes was 94.1%, there were no half-gravid or gravid specimens collected. The absence of half-gravid and gravid mosquitoes collected in both the EWT and indoor resting surveys indicates that *An. farauti *is a highly exophilic vector, leaving the house on the night of feeding.

### Detection of circumsporozoite antigen and entomological inoculation rates

From the 2008 pre-intervention collections in Pala, 2,561 *An. farauti *were tested for circumsporozoite antigen and all were negative. In Lata, 1,150 *An. farauti *were tested with three specimens confirmed positive for *P. vivax *(247 var. antigen). The sporozoite rate for Lata was 0.0026 (Table 2). The entomological inoculation rate (EIR) was calculated as 18.25 infective bites/person/year (ib/p/y). In the post-intervention survey of April 2010, the *P. vivax *(247 var. antigen) sporozoite rate of *An. farauti *in Pala was 0.0013 (*n = *749 specimens assayed) representing an EIR of 10.74 ib/p/y. In June 2010, 1,308 *An. farauti*, collected in Pala, were assayed, two were found positive for *P. vivax *(247 var. antigen) circumsporozoite antigen indicating a sporozoite rate of 0.0015 (Table 2). From post-intervention collections made in Pala in December 2010 1,292 *An. farauti *were assayed with one specimen found positive for *P. vivax *(247 var. antigen) circumsporozoite antigen, indicating a sporozoite rate of 0.0008 and an EIR of 17.59 ib/p/y (Table 2).

### Alternative hosts

The alternative hosts present in Temotu villages were pigs and dogs; there were no cattle, buffalo, or horses. The number pigs and dogs in Pala were 21 and 17 respectively with 62% of household owning pigs and 38% owning dogs. In Lata there were 46 pigs and 33 dogs and 46% of households owned pigs and 41% owned dogs. Pigs were kept on the periphery of the village in Pala and throughout the village in Lata. Dogs lived within the household. All animals were accessible to *An. farauti *seeking a bloodmeal with in the vicinity of the village. However, no *An. farauti *mosquitoes were collected from inside of pig pens at 6 am.

## Discussion

In the Solomon Islands, the malaria eradication programme of the early 1970's achieved considerable success against malaria transmission using DDT-based IRS [17]. This intervention measure was particularly effective against two of the malaria vectors, *Anopheles punctulatus *and *Anopheles koliensis*, as these two species were late night biting, endophagic species and virtually disappeared after the first few spray rounds [33,34]. However, ultimately eradication was not attained primarily due to several factors. In addition to the logistic and financial limitations that might also account the failure of the malaria eradication of the 1970s, the change in behaviour of the third vector *Anopheles farauti *which, due to the irritant effect of the insecticide, avoided entering sprayed houses and maintained outdoor biting populations by feeding early in the evening [8,18]. This was possible as Melanesian villages frequently spend the early part of the evening outdoors. Even though *An. farauti *can be quite indiscriminate in its host preference [9], in the shift to outdoor early night feeding to avoid the IRS no major shift to zoophilia has been observed in either the Solomon Islands [3] or Papua New Guinea [15]. Following the eradication programme, IRS use was continued as a control tool until insecticide treated nets were introduced in the early 1990s [35]. Since then, these intervention measures, plus improved health infrastructure and education, has seen malaria slowly decline from a peak in 1992 [4].

In Temotu Province, where malaria elimination is currently being implemented, this study confirmed that *An. farauti *was the only anopheline in the province and thus the vector of malaria. In 2008, at the commencement of the elimination programme, there was some heterogeneity in biting patterns observed between localities, but this species was generally an early night biter and primarily exophagic and exophilic. Comparative historical biting patterns for *An. farauti *in Santa Cruz, Temotu are lacking from the published literature. However, research conducted in nearby Makira Island by Taylor [8] in 1971-73 presents an indication of the natural biting behaviour of *An. farauti*. Without exposure to any insecticide pressure, about half (53.8%) of the *An. farauti *population bites humans indoors and although the peak biting activity is fairly early in the evening (7 pm - 8 pm), there is consistent biting activity until midnight after which biting slowly waned [8]. The biting profiles observed in Temotu in 2008 were altered from this historical reference with a pronounced shift to early night feeding and outdoor biting a phenomena that was seen with *An. farauti *throughout the Solomon Islands following the introduction of the eradication programmes [17,18] and which has probably been maintained over the years since then by the continuous low level of vector control implemented on the island.

By comparing data obtained in Pala village from 2008 to 2010, it is evident that two rounds of pyrethroid IRS and LLIN use was able to further alter the biting behaviour of the mosquito population. After only 2 years of intense vector control, the proportion of human contact occurring indoors dropped from 57.0% to 36.7 - 40.4%. The mechanism underlying this change in behaviour is thought to be the vectors response to the irritability of the insecticide. It is feasible that the prolonged and wide-spread use of IRS and LLINs would favour individual survival traits such as biting outdoors or early in the evening, and with time these traits may be selected for phenotypically and genetically. While the development of behavioural avoidance is a consequence of well applied and effective vector control measures it also highlights that IRS and LLINs, which target an indoor feeding vector, will be insufficient to eliminate malaria in a range of ecological settings [36,37].

It has been previously demonstrated that wide-spread use of IRS and/or LLINs is able to reduce the density, feeding frequency and survival (as measured by parity ratios) of the vector at the population level by reducing vector densities and human vector contact [3,35,38-41]. However, the current study did not observe a reduction in any of these classical individual-level indicators of insecticide use. While this study observed fluctuations in mosquito densities, there was no sustained drop in human biting rates in Pala. There was no reduction in vector longevity with the proportion of parous mosquitoes remaining around 42% pre- and post-intervention. Also the sporozoite rate and EIR indicated that there was a low, but persistent, level of transmission still continuing two years after the intervention measures were implemented (Table 2).

So the intervention measures appear to have had little impact on the density and longevity of the vector. These finding beg the question that why the malaria rates are so low in Temotu Province, and why have they been falling over the last decade? [4]. It may be that in Temotu Province, *An. farauti *is not a particularly efficient vector of malaria, as mentioned it is not a particularly long lived mosquito with only a small fraction of the population living long enough to transmit the disease. In other parts of its range, estimates of parity rates have been considerable higher, ranging from 52% - 72% [3,14,15,35,42].

With the change in the feeding behaviour of *An. farauti *compromising the malaria intervention measures there will be a need to supplement these measures with other elimination tools which don't rely on late night indoor feeding behaviour of the adults. The surveys conducted here indicated that large productive breeding sites, such as coastal swamps, were responsible for the high vector densities observed initially in Pala and Lata and that the elimination of the swamp at Lata effectively eliminated the adult biting population. As these swamps are not common and easy to locate further studies are warranted to determine if larviciding of these sites could be used as a supplement to the measures already in place against the adults. On the other hand, alternative hosts were relatively available with up to 62% of households owning pigs. This availability of alternative hosts coupled with published literature from Papua New Guinea indicating that *An. farauti *will feed on such hosts [15,32], indicates that zooprophylaxis could be a potential tool for Solomon Islands.

## Conclusion

With an inefficient vector, any intervention measures, even sub-optimal ones, will have some impact on malaria transmission [27]. However, if the goal for Temotu Province is malaria elimination additional intervention measures will be needed. Developing integrated vector control programmes will require the use of complementary strategies that consider the subtleties of mosquito ecology to further reduce the density of the local vector population. The surveys conducted here indicate that the most productive *An. farauti *breeding sites are large permanent swamps and when such sites are removed *An. farauti *densities drop dramatically. These sites are restricted to the coast, overt and not that common and could be readily treated with a long lasting larvicide. As such, incorporating larval control and/or zooprophylaxis into the programme control might further suppress malaria transmission by supplementing the tools that are already in place to target the adult.

## Competing interests

The authors declare that they have no competing interests.

## Authors' contributions

Conceived and designed the experiments: HB, RDC. Performed the experiments: HB, RDB, CB, CI, TLR. Performed the molecular analysis: DOM. Analysed the data and wrote the manuscript: HB, RDC, TLR. Reviewed the manuscript: DOM, CCC. All authors have read and approved the final manuscript.
